# (2*E*)-2-Benzyl­idene-*N*-phenyl­hydrazinecarboxamide

**DOI:** 10.1107/S1600536814008344

**Published:** 2014-04-26

**Authors:** S. R. Layana, M. Sithambaresan, V. L. Siji, M. R. Sudarsanakumar, S. Suma

**Affiliations:** a Department of Chemistry, Mahatma Gandhi College, Thiruvananthapuram 695 004, Kerala, India; b Department of Chemistry, Faculty of Science, Eastern University, Sri Lanka, Chenkalady, Sri Lanka; c Department of Chemistry, All Saints College, Thiruvananthapuram 695 007, Kerala, India; d Department of Chemistry, Sree Narayana College, Chempazhanthy, Thiruvananthapuram 695 587, Kerala, India

## Abstract

The mol­ecule of the title compound, C_14_H_13_N_3_O, adopts an *E* conformation with respect to the azomethine C=N bond, and is roughly planar, with an r.m.s. deviation of the non-H atoms from the least-squares plane of 0.100 (2) Å and a dihedral angle between the terminal benzene rings of 5.74 (12)°. An intramolecular N—H⋯N hydrogen bond closes an *S*(6) ring. In the crystal, mol­ecules are linked by the pairs of N—H⋯O hydrogen bonds into centrosymmetric dimers. Dimers related by translation along [010] form slanted stacks, the shortest C⋯C inter­molecular distance within the stack being 3.283 (3) Å. Weak C—H⋯π inter­actions link the stacks into a three-dimensional structure.

## Related literature   

For the synthesis of related compounds, see: Siji *et al.* (2010 ▶). For biological applications of hydrazinecarboxamide and its derivatives, see: Rivadeneira *et al.* (2009 ▶); Shalini *et al.* (2009 ▶). For related structures, see: Annie *et al.* (2012 ▶); Aravindakshan *et al.* (2013 ▶).
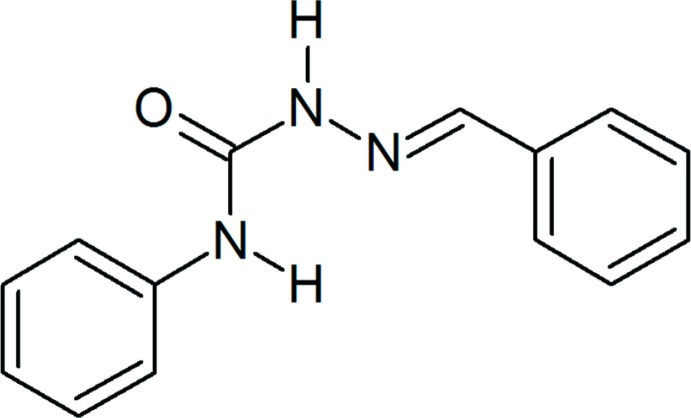


## Experimental   

### 

#### Crystal data  

C_14_H_13_N_3_O
*M* *_r_* = 239.27
Monoclinic, 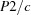

*a* = 13.6308 (14) Å
*b* = 5.4023 (5) Å
*c* = 17.5751 (19) Å
β = 93.065 (4)°
*V* = 1292.3 (2) Å^3^
*Z* = 4
Mo *K*α radiation
μ = 0.08 mm^−1^
*T* = 296 K
0.29 × 0.24 × 0.21 mm

#### Data collection  

Bruker APEXII CCD area-detector diffractometer
Absorption correction: multi-scan (*SADABS*; Bruker, 2004 ▶) *T*_min_ = 0.977, *T*_max_ = 0.983
9857 measured reflections
2302 independent reflections
1655 reflections with *I* > 2σ(*I*)
*R*_int_ = 0.023

#### Refinement  

*R*[*F* ^2^ > 2σ(*F* ^2^)] = 0.044
*wR*(*F* ^2^) = 0.146
*S* = 1.04
2300 reflections
171 parameters
2 restraints
H atoms treated by a mixture of independent and constrained refinement
Δρ_max_ = 0.19 e Å^−3^
Δρ_min_ = −0.14 e Å^−3^

### 

Data collection: *APEX2* (Bruker, 2004 ▶); cell refinement: *SAINT* (Bruker, 2004 ▶); data reduction: *SAINT*; program(s) used to solve structure: *SHELXS97* (Sheldrick, 2008 ▶); program(s) used to refine structure: *SHELXL97* (Sheldrick, 2008 ▶); molecular graphics: *ORTEP-3 for Windows* (Farrugia, 2012 ▶) and *Mercury* (Macrae *et al.*, 2008 ▶); software used to prepare material for publication: *SHELXL97* and *publCIF* (Westrip, 2010 ▶).

## Supplementary Material

Crystal structure: contains datablock(s) I, global. DOI: 10.1107/S1600536814008344/yk2104sup1.cif

Structure factors: contains datablock(s) I. DOI: 10.1107/S1600536814008344/yk2104Isup2.hkl

Supporting information file. DOI: 10.1107/S1600536814008344/yk2104Isup3.cml

CCDC reference: 997059

Additional supporting information:  crystallographic information; 3D view; checkCIF report

## Figures and Tables

**Table 1:** Hydrogen-bond geometry (Å, °) *Cg*1 is the centroid of the C9–C14 ring.

*D*—H⋯*A*	*D*—H	H⋯*A*	*D*⋯*A*	*D*—H⋯*A*
N3—H3′⋯N1	0.88 (1)	2.20 (2)	2.634 (2)	110 (2)
N2—H2′⋯O1^i^	0.88 (1)	2.00 (1)	2.860 (2)	167 (2)
C3—H3⋯*Cg*1^ii^	0.93	2.99	3.800 (3)	146

Symmetry codes: (i) 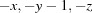; (ii) 
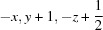
.

**Fig. 1.**
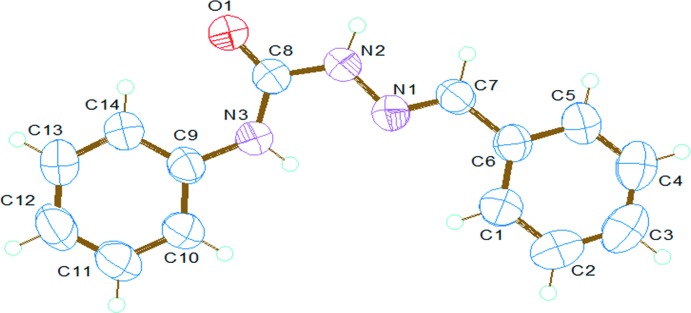
ORTEP view of the compound, drawn with 50% probability displacement ellipsoids for the non-H atoms.

**Fig. 2.**
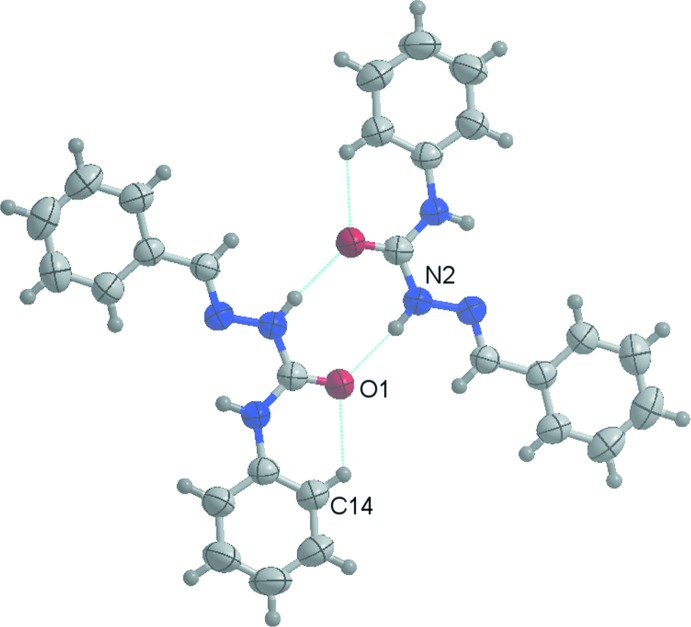
Centrosymmetric dimer formed by pair of hydrogen bonds in the title compound.

**Fig. 3.**
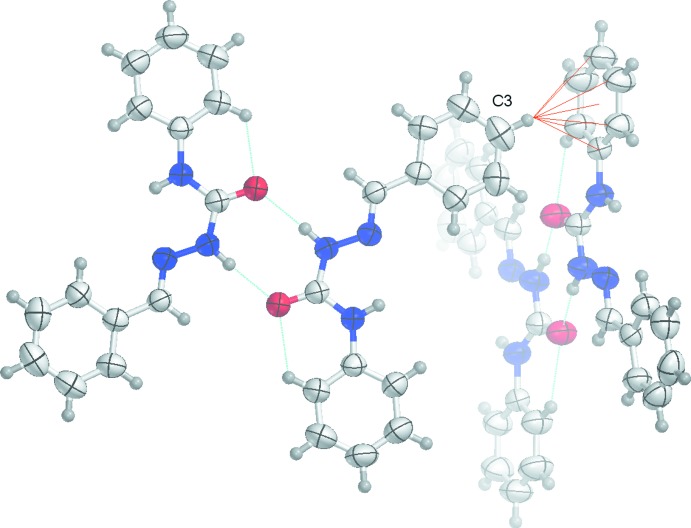
C—H···π interaction in the title compound.

**Fig. 4.**
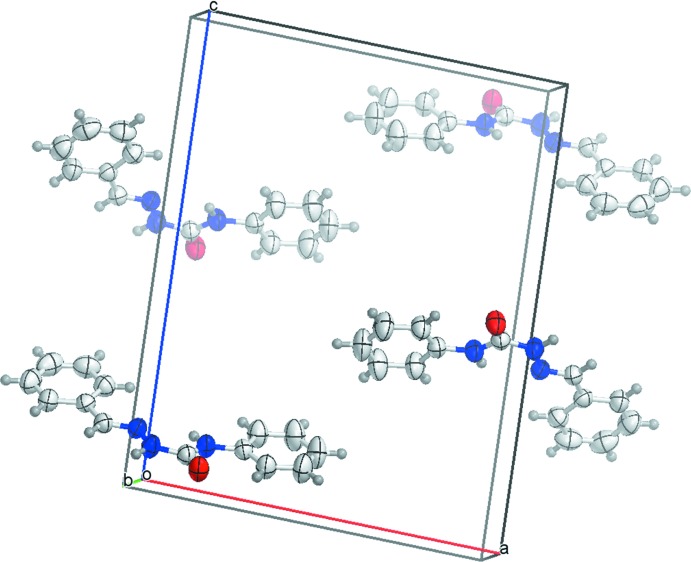
A view of the unit cell along b axis.

## supplementary crystallographic information

### 1. Comment

Hydrazinecarboxamides with versatile structural features are good ligands for both the neutral and anionic complexes (Rivadeneira *et al.*, 2009). The aryl hydrazinecarboxamides were found to be devoid of sedative hypnotic activity and exhibited anticonvulsant activity with less neurotoxicity (Shalini *et al.*, 2009).

The title compound (Scheme 1, Fig. 1) crystallizes in the monoclinic space group *P*2/*c*. The molecule adopts an *E* configuration with respect to C7═N1 bond and the N3–C8–N2–N1 torsion angle of 4.3 (3)° reveals that N1 and N3 atoms are *cis* to each other with respect to C8—N2 bond. The central fragment N3–C8(O1)–N2–N1 is almost planar and forms diherdal angles with two benzene rings of 13.24 (11)° (C1—C6) and of 7.60 (12)° (C9—C14).

The C7—N1 (1.273 (2) Å) and C8—O1 (1.225 (2) Å) bond distances are very close to the reported bond lengths of azomethine and keto groups, respectively, in similar structures (Annie *et al.*, 2012), confirming the azomethine bond formation and existence of semicarbazone in amido form. A five-membered ring is formed by N1/N2/C8/N3 and H3' through an intramolecular H-bonding interaction with a D···A distance of 2.634 Å. In the crystal, a centrosymmetric dimer (Aravindakshan *et al.*, 2013) is formed by means of a classical intermolecular N—H···O hydrogen bond (Fig. 2) with a D···A distance of 2.860 (2) Å. A C—H···π interaction (Fig. 3) is present in the crystal between the hydrogen attached to the carbon C3 and the C9—C14 benzene ring with H···π distance of 2.99 Å, interconnects the adjacent centrosymmetric dimers to build a three-dimensional supramolecular architecture in the system. Fig. 4 shows the packing diagram of the title compound along *b* axis.

### 2. Experimental

The title compound was prepared by adapting a reported procedure (Siji *et al.*, 2010). Hot methanolic solutions (25 ml) of equimolar amounts of benzaldehyde (0.106 g, 1 mmol) and *N*-phenylhydrazinecarboxamide (0.151 g, 1 mmol) were mixed and refluxed for 3 h after adding a few drops of dilute acetic acid. The resulting solution was cooled to room temperature. The colourless block shaped crystals were collected, washed with few drops of methanol and dried over P_4_O_10_*in vacuo*. Single crystals suitable for X-ray analysis were obtained by slow evaporation of solution in air for few days.

IR (KBr, υ in cm^-1^): 3356, 2959, 1684, 1540, 1024. ^1^H NMR (400 MHz, CDCl_3_, δ, p.p.m.): 9.51 (s, 1H), 8.13 (s, 1H), 7.85 (s, 1H), 7.09–7.58 (m, 5H), 7.09–7.67 (m, 10H). ^13^C NMR (400 MHz, CDCl_3_, δ, p.p.m.): 153.61, 141.78, 137.84, 133.66, 130.02, 129.02, 128.79, 126.94, 123.54, 119.68.

### 3. Refinement

All H atoms on C were placed in calculated positions, guided by difference maps, with C—H bond distances of 0.93 Å. H atoms were assigned *U*_iso_(H) values of 1.2Ueq(carrier). H atoms of N3—H3' and N2—H2' bonds were located from difference maps and the bond distances are restrained to 0.88±0.01 Å.

### Crystal data

**Table:**

C_14_H_13_N_3_O	*F*(000) = 504
*M**_r_* = 239.27	*D*_x_ = 1.230 Mg m^−^^3^
Monoclinic, *P*2/*c*	Mo *K*α radiation, λ = 0.71073 Å
Hall symbol: -P 2yc	Cell parameters from 3449 reflections
*a* = 13.6308 (14) Å	θ = 2.7–25.1°
*b* = 5.4023 (5) Å	µ = 0.08 mm^−^^1^
*c* = 17.5751 (19) Å	*T* = 296 K
β = 93.065 (4)°	Block, colourless
*V* = 1292.3 (2) Å^3^	0.29 × 0.24 × 0.21 mm
*Z* = 4	

### Data collection

**Table:**

Bruker APEXII CCD area-detector diffractometer	2302 independent reflections
Radiation source: fine-focus sealed tube	1655 reflections with *I* > 2σ(*I*)
Graphite monochromator	*R*_int_ = 0.023
Detector resolution: 8.33 pixels mm^-1^	θ_max_ = 25.1°, θ_min_ = 2.3°
φ and ω scans	*h* = −16→16
Absorption correction: multi-scan (*SADABS*; Bruker, 2004)	*k* = −6→6
*T*_min_ = 0.977, *T*_max_ = 0.983	*l* = −20→20
9857 measured reflections	

### Refinement

**Table:**

Refinement on *F*^2^	Primary atom site location: structure-invariant direct methods
Least-squares matrix: full	Secondary atom site location: difference Fourier map
*R*[*F*^2^ > 2σ(*F*^2^)] = 0.044	Hydrogen site location: inferred from neighbouring sites
*wR*(*F*^2^) = 0.146	H atoms treated by a mixture of independent and constrained refinement
*S* = 1.04	*w* = 1/[σ^2^(*F*_o_^2^) + (0.0686*P*)^2^ + 0.3096*P*] where *P* = (*F*_o_^2^ + 2*F*_c_^2^)/3
2300 reflections	(Δ/σ)_max_ < 0.001
171 parameters	Δρ_max_ = 0.19 e Å^−^^3^
2 restraints	Δρ_min_ = −0.14 e Å^−^^3^

### Special details

**Table:**

Geometry. All e.s.d.'s (except the e.s.d. in the dihedral angle between two l.s. planes) are estimated using the full covariance matrix. The cell e.s.d.'s are taken into account individually in the estimation of e.s.d.'s in distances, angles and torsion angles; correlations between e.s.d.'s in cell parameters are only used when they are defined by crystal symmetry. An approximate (isotropic) treatment of cell e.s.d.'s is used for estimating e.s.d.'s involving l.s. planes.
Refinement. Refinement of *F*^2^ against ALL reflections. The weighted *R*-factor *wR* and goodness of fit *S* are based on *F*^2^, conventional *R*-factors *R* are based on *F*, with *F* set to zero for negative *F*^2^. The threshold expression of *F*^2^ > σ(*F*^2^) is used only for calculating *R*-factors(gt) *etc*. and is not relevant to the choice of reflections for refinement. *R*-factors based on *F*^2^ are statistically about twice as large as those based on *F*, and *R*- factors based on ALL data will be even larger.

### Fractional atomic coordinates and isotropic or equivalent isotropic displacement parameters (Å^2^)

**Table:**

	*x*	*y*	*z*	*U*_iso_*/*U*_eq_	
O1	0.12555 (9)	−0.4737 (3)	0.03527 (9)	0.0743 (4)	
N1	−0.03540 (11)	−0.0322 (3)	0.10278 (9)	0.0613 (4)	
N2	−0.00160 (11)	−0.2374 (3)	0.06671 (10)	0.0678 (5)	
N3	0.15497 (11)	−0.1014 (3)	0.09396 (10)	0.0642 (4)	
C1	−0.12124 (15)	0.3641 (4)	0.18518 (12)	0.0718 (6)	
H1	−0.0535	0.3456	0.1924	0.086*	
C2	−0.1678 (2)	0.5526 (4)	0.22135 (14)	0.0879 (7)	
H2	−0.1315	0.6601	0.2532	0.105*	
C3	−0.2675 (2)	0.5831 (5)	0.21077 (16)	0.0924 (8)	
H3	−0.2989	0.7108	0.2353	0.111*	
C4	−0.32004 (18)	0.4260 (5)	0.16432 (16)	0.0885 (7)	
H4	−0.3875	0.4474	0.1567	0.106*	
C5	−0.27437 (14)	0.2354 (4)	0.12843 (12)	0.0735 (6)	
H5	−0.3114	0.1278	0.0972	0.088*	
C6	−0.17410 (13)	0.2018 (3)	0.13822 (10)	0.0585 (5)	
C7	−0.12822 (13)	−0.0028 (4)	0.09942 (11)	0.0605 (5)	
H7	−0.1677	−0.1145	0.0715	0.073*	
C8	0.09629 (13)	−0.2821 (4)	0.06361 (11)	0.0596 (5)	
C9	0.25809 (13)	−0.0996 (3)	0.10287 (11)	0.0593 (5)	
C10	0.29986 (15)	0.0908 (4)	0.14580 (15)	0.0811 (7)	
H10	0.2601	0.2115	0.1659	0.097*	
C11	0.40000 (17)	0.1028 (5)	0.15888 (18)	0.0985 (8)	
H11	0.4275	0.2300	0.1886	0.118*	
C12	0.45930 (17)	−0.0700 (5)	0.12867 (19)	0.1017 (9)	
H12	0.5271	−0.0598	0.1370	0.122*	
C13	0.41822 (16)	−0.2588 (5)	0.08601 (17)	0.0977 (8)	
H13	0.4587	−0.3768	0.0653	0.117*	
C14	0.31741 (14)	−0.2772 (4)	0.07315 (13)	0.0773 (6)	
H14	0.2901	−0.4079	0.0448	0.093*	
H2'	−0.0434 (12)	−0.336 (3)	0.0420 (11)	0.075 (6)*	
H3'	0.1243 (14)	0.026 (3)	0.1121 (12)	0.078 (7)*	

### Atomic displacement parameters (Å^2^)

**Table:**

	*U*^11^	*U*^22^	*U*^33^	*U*^12^	*U*^13^	*U*^23^
O1	0.0593 (8)	0.0682 (9)	0.0949 (11)	0.0025 (6)	−0.0021 (7)	−0.0228 (7)
N1	0.0535 (9)	0.0662 (10)	0.0646 (10)	−0.0012 (7)	0.0064 (7)	−0.0066 (7)
N2	0.0501 (9)	0.0714 (11)	0.0820 (11)	−0.0038 (8)	0.0036 (8)	−0.0216 (9)
N3	0.0520 (9)	0.0623 (10)	0.0776 (11)	0.0030 (7)	−0.0022 (7)	−0.0141 (8)
C1	0.0687 (12)	0.0747 (14)	0.0718 (13)	0.0010 (10)	0.0009 (10)	−0.0056 (10)
C2	0.112 (2)	0.0756 (15)	0.0759 (15)	−0.0007 (13)	0.0062 (13)	−0.0110 (12)
C3	0.112 (2)	0.0763 (15)	0.0919 (18)	0.0256 (14)	0.0291 (15)	0.0068 (13)
C4	0.0737 (14)	0.0932 (17)	0.0995 (18)	0.0234 (13)	0.0137 (13)	0.0107 (14)
C5	0.0582 (11)	0.0841 (15)	0.0783 (14)	0.0038 (10)	0.0046 (10)	0.0023 (11)
C6	0.0564 (10)	0.0630 (11)	0.0566 (11)	−0.0001 (8)	0.0072 (8)	0.0048 (9)
C7	0.0528 (10)	0.0684 (12)	0.0603 (11)	−0.0057 (8)	0.0018 (8)	−0.0053 (9)
C8	0.0532 (10)	0.0630 (11)	0.0623 (11)	−0.0005 (9)	0.0009 (8)	−0.0040 (9)
C9	0.0517 (10)	0.0605 (11)	0.0651 (11)	0.0007 (8)	−0.0017 (8)	0.0039 (9)
C10	0.0625 (12)	0.0685 (13)	0.1114 (18)	−0.0038 (10)	−0.0036 (11)	−0.0131 (12)
C11	0.0678 (14)	0.0837 (16)	0.142 (2)	−0.0114 (12)	−0.0128 (14)	−0.0153 (15)
C12	0.0534 (12)	0.0997 (19)	0.151 (3)	−0.0060 (13)	−0.0056 (14)	−0.0021 (17)
C13	0.0603 (13)	0.1020 (19)	0.131 (2)	0.0146 (13)	0.0054 (13)	−0.0146 (16)
C14	0.0589 (11)	0.0817 (14)	0.0909 (15)	0.0045 (10)	0.0001 (10)	−0.0161 (12)

### Geometric parameters (Å, º)

**Table:**

O1—C8	1.225 (2)	C4—H4	0.9300
N1—C7	1.273 (2)	C5—C6	1.380 (3)
N1—N2	1.369 (2)	C5—H5	0.9300
N2—C8	1.360 (2)	C6—C7	1.457 (3)
N2—H2'	0.877 (9)	C7—H7	0.9300
N3—C8	1.353 (2)	C9—C14	1.376 (3)
N3—C9	1.406 (2)	C9—C10	1.380 (3)
N3—H3'	0.876 (9)	C10—C11	1.373 (3)
C1—C2	1.374 (3)	C10—H10	0.9300
C1—C6	1.380 (3)	C11—C12	1.361 (4)
C1—H1	0.9300	C11—H11	0.9300
C2—C3	1.373 (4)	C12—C13	1.368 (4)
C2—H2	0.9300	C12—H12	0.9300
C3—C4	1.355 (4)	C13—C14	1.384 (3)
C3—H3	0.9300	C13—H13	0.9300
C4—C5	1.374 (3)	C14—H14	0.9300
			
C7—N1—N2	115.97 (15)	N1—C7—C6	121.55 (17)
C8—N2—N1	121.18 (15)	N1—C7—H7	119.2
C8—N2—H2'	119.0 (13)	C6—C7—H7	119.2
N1—N2—H2'	119.7 (13)	O1—C8—N3	124.84 (16)
C8—N3—C9	127.98 (16)	O1—C8—N2	120.54 (16)
C8—N3—H3'	115.4 (14)	N3—C8—N2	114.62 (17)
C9—N3—H3'	116.6 (14)	C14—C9—C10	119.58 (18)
C2—C1—C6	120.6 (2)	C14—C9—N3	123.85 (17)
C2—C1—H1	119.7	C10—C9—N3	116.54 (17)
C6—C1—H1	119.7	C11—C10—C9	120.2 (2)
C3—C2—C1	120.3 (2)	C11—C10—H10	119.9
C3—C2—H2	119.8	C9—C10—H10	119.9
C1—C2—H2	119.8	C12—C11—C10	120.5 (2)
C4—C3—C2	119.6 (2)	C12—C11—H11	119.7
C4—C3—H3	120.2	C10—C11—H11	119.7
C2—C3—H3	120.2	C11—C12—C13	119.4 (2)
C3—C4—C5	120.6 (2)	C11—C12—H12	120.3
C3—C4—H4	119.7	C13—C12—H12	120.3
C5—C4—H4	119.7	C12—C13—C14	121.1 (2)
C4—C5—C6	120.7 (2)	C12—C13—H13	119.4
C4—C5—H5	119.6	C14—C13—H13	119.4
C6—C5—H5	119.6	C9—C14—C13	119.1 (2)
C1—C6—C5	118.23 (19)	C9—C14—H14	120.4
C1—C6—C7	122.55 (17)	C13—C14—H14	120.4
C5—C6—C7	119.22 (18)		
			
C7—N1—N2—C8	177.05 (17)	C9—N3—C8—N2	176.29 (18)
C6—C1—C2—C3	−0.5 (3)	N1—N2—C8—O1	175.52 (17)
C1—C2—C3—C4	−0.1 (4)	N1—N2—C8—N3	−4.3 (3)
C2—C3—C4—C5	0.7 (4)	C8—N3—C9—C14	7.8 (3)
C3—C4—C5—C6	−0.8 (3)	C8—N3—C9—C10	−170.6 (2)
C2—C1—C6—C5	0.4 (3)	C14—C9—C10—C11	−0.1 (4)
C2—C1—C6—C7	−179.21 (19)	N3—C9—C10—C11	178.4 (2)
C4—C5—C6—C1	0.2 (3)	C9—C10—C11—C12	1.2 (4)
C4—C5—C6—C7	179.83 (18)	C10—C11—C12—C13	−1.1 (5)
N2—N1—C7—C6	177.24 (16)	C11—C12—C13—C14	−0.1 (4)
C1—C6—C7—N1	−4.9 (3)	C10—C9—C14—C13	−1.0 (3)
C5—C6—C7—N1	175.51 (18)	N3—C9—C14—C13	−179.4 (2)
C9—N3—C8—O1	−3.5 (3)	C12—C13—C14—C9	1.1 (4)

### Hydrogen-bond geometry (Å, º)

Cg1 is the centroid of the C9–C14 ring.

**Table:**

*D*—H···*A*	*D*—H	H···*A*	*D*···*A*	*D*—H···*A*
N3—H3′···N1	0.88 (1)	2.20 (2)	2.634 (2)	110 (2)
N2—H2′···O1^i^	0.88 (1)	2.00 (1)	2.860 (2)	167 (2)
C3—H3···*Cg*1^ii^	0.93	2.99	3.800 (3)	146

Symmetry codes: (i) −*x*, −*y*−1, −*z*; (ii) −*x*, *y*+1, −*z*+1/2.
